# Dress Reform

**Published:** 1875-08

**Authors:** 


### DRESS REFORM.

A committee of the Dress Reform Association, composed of earnest ladies of high-standing in Boston, tried the experiment a year or more since, of opening a store for the sale of sensible clothing for ladies. These good women have made the study of DRESS a subject of profound thought, and determined that there was great room for improvement. They believed, too, that the women of New England would second their efforts in the direction of reform. So they struck out boldly in the manufacture of ladies' wardrobes, upon these principles, viz: First, that the vital organs in central regions of the body should be allowed unimpeded action. Second, that a uniform temperature of the body should be preserved. Third, that weight should be reduced to a minimum. Fourth, that the shoulders, and not the hips, should form the base of support.

The result of this organization, based upon these principles, has been a complete success. So many calls have come from New York and vicinity for these garments, that the committee have decided to open a similar establishment in New York, and have accordingly taken the spacious first floor of No. 16 West Fourteenth street, which they are elaborately fitting up and which they will fully open for business on the first day of September. The rooms will be in charge of the Dress Reform Committee, at the head of which is Mrs. H. S. Hutchinson, whose intelligence and tact have rendered the Boston work so largely successful.

We congratulate our friends in this vicinity as well as those in more distant parts, upon the fact which we have here announced, and will, from time to time, describe the new and better articles of apparel which this committee introduce for the benefit ot those who suffer injury and deformity from the vicious clothing so largely worn.

### CHOLERA INFANTUM.

We see it stated that Cholera Infantum is raging with great violence in Baltimore, and that infant mortality is just now very large on account of this dreaded disease. Usually, children who are well-fed, well-housed, well-bathed, and otherwise well-cared for, are comparatively exempt from the ravages of this disease. The chances are rather against any other class. But we have found no difficulty in saving all the numerous cases which we have been called to, since we began to use the prescription which is now put up in little sugar pelets, as Van Deren's Remedy. We believe we have been the Providential instrument in saving over 2000 little lives with that remedy alone, added to good nursing and proper food. We will stake our medical reputation on its success in all reasonable cases. One hundred children will die this summer from bowel-complaints, when not more than one would die, if this remedy were given. We say this from a purely disinterested and humanitarian stand-point, and do not hesitate to affirm that if all the Editors of America felt as we do, and were as anxious as we are to save the young lives of those who may become useful men and women, they would make haste to copy this little item.

---

HOW TO SWEEP A CARPET.—The right way to sweep is to incline the handle of the broom a little forward, then give a light drawing stroke, allowing the broom to scarcely touch the carpet. Not half the weight of the broom should be allowed to press on the carpet as the dirt is moved forward. Let the dirt be moved and rolled lightly. If a generous quantity of tea-grounds or clean and damp sawdust can be spread over the carpet before sweeping, all the fine dirt will adhere to the wet materials.

### GRANULATED WHEAT BISCUIT.

An ingenious gentleman, residing in the interior of the State of New York, has conceived the idea that a much more palatable and nutritious article of food can be fabricated from wheat, than has heretofore been made. He has carried his ideas into effect, with quite remarkable results.

The chief features of his improved system are the means employed in the reduction of the grain to powder, and in the mixing and baking processes. In these items the change is radical.

He does not grind his wheat in the ordinary way, but pounds it with hammers, run by steam power. The grain runs through a "stamp-mill," similar to that used for crushing ores in mining countries.

To obviate the tendency in the ordinary wheat, to heat and "gum up," it is first dried in an oven, "kiln-dried," as it were. This prepares it for crushing, when a blow breaks the kernel and reduces it to a dry powder at once. If it is desired to make a fine and very white biscuit, the powdered wheat is sifted or "bolted," in the usual way; if, on the contrary, a darker biscuit, containing all the valuable contents of the cereal, is preferred, the powder is not subjected to any sifting process. In either event, the subsequent processes are the same.

The powder, or flour, is placed in huge troughs in which great arms ply back and forth, actuated by steam power. Water, pure and cool, is added, a little at a time, and the arms are set in motion.

By degrees, the mass becomes a stiff dough. When thoroughly mixed this dough is placed under heavy hammers, where it receives a tremendous pounding. The effect of this is to make the dough whiter in color and lighter in consistence. From a heavy, agglutinated mass, it becomes, after an hours hard beating, a light, porous and sponge-like substance. From the beating trough it passes between ponderous polished rollers, directly to the cutting-machine. The rollers having reduced it to a very thin sheet, the cutters divide the sheet into round disks or wafers, which are conveyed by an endless chain into a soap-stone oven, of peculiar construction, where they are baked.

In the whole process, the materials are not touched by human hands. This is a feature which cannot be to highly commended. The work of the ordinary baker is one of great heat, and is of course attended with profuse perspiration . It is horrible to reflect that the most hateful secretions of the human body, the exudations from those true sewers of the system—the perspiratory ducts—saturated with the waste and poison of the body—it is horrible to reflect that this poisonous matter, which would destroy life if not thrown off, is mingled with nine-tenths of all the bread we eat! The disease thus induced will never be computed, because the vastness of the evil can scarcely be comprehended. That a bread, or wheat product, at last exists which is free from this fearful contamination of the sweat of human bodies, is a consolation indeed.

### ACTION OF MEDICINES.

BY E. H. GIBBS, A.M., M.D.

The immediate effect of nearly all medicines is to stimulate the vital organs, to increased action. The ultimate result of such stimulation is fatigue or prostration. Such are the results of what are known as "simple cathartics." There are other medicines that have a specific action upon the blood, producing chemical changes which destroy diseases dependent upon infection; and when wisely and cautiously administered do not destroy the *patient.*

All preparations of opium stimulate at first; but the stimulation is usually followed by the most depressing reaction. Opiates should be employed with great caution. When thus employed they are—all things considered—the greatest boon to suffering humanity of all the medicines known to man.

On account of the prompt and decided action of opium, it is extensively used in the preparation of certain nostrums with which the country is flooded and the people afflicted. There is no "soothing syrup" that does not contain it in dangerous quantities; no cough syrup the virtues of which do not depend on this poison.

The effect of opium in all its forms, upon young children, is simply terrible. Nothing but a plea of the grossest ignorance should shield a parent or guardian from severe punishment, who would ever permit a particle of opium to be given to a child under twelve years of age. It acts with fourfold more power on children than upon adults. In all cases when given to children, its effect is directly upon the liver; at first impairing its action and finally destroying its function. Thousands of young children are sacrificed in the United States every summer,—not by Cholera Infantum or Summer Complaint, as is generally supposed,—but by *opium.* As summer approaches, the little victims are called out to make up the usual statistics of this cruel and useless slaughter. Heaven help them till a more enlightened public opinion, or wise Legislative Enactments come to their rescue. It is sad to think of the sacrifices that continue each season to be made to a disease that almost invariably yields to proper treatment. "Cholera Infantum and Summer Complaint of children," was the subject of a previous article in this journal.

Tonics, in small doses stimulate. But their effect upon the system is so gradual that there is no appreciable reaction. Quinine is the most potent of all tonics that are classed as medicines. Although its effect is almost specific in certain cases, its mode of action is a mystery.

"A discussion of the "*modus operandi,*" with our present knowledge would avail nothing. Iron is another tonic, the effect of which on the system is more satisfactorily explained. Debility is frequently caused by a deficiency of iron in the blood. This deficiency may be supplied by tonics containing iron, when the usual health is restored.

But the finest tonics are prepared in nature's laboratory; these are sunshine and fresh air. Supplement them with wholesome food, exercise, and a contented spirit, and health and long life will be the reward.

### THE BEST FOOD.

As a rule, not only is the simplest food the best food, but the most seasonable is, in the long run, the most appetizing. There is no difficulty in determining what we should eat, since the products of our climate show us plainly month by month. Fish, flesh, and fruit, by their plumpness, tenderness, and ripeness, themselves denote when they are ready to be eaten. A sound stomach will profit by whatever an unspoiled palate enjoys.

The wholesomeness of food depends nearly as much on the time it is taken as on the quantity. We have grown so luxurious in our physical as well as mental tastes, that we are constantly tempted to eat things out of season. Yielding to the temptation, as we often do, we pay the penalty, soon or late, in temporary or chronic derangement of our health. The meat which is excellent in cold, may not be desirable iu warm weather; fish is best during Spring and early Summer; vegetables and fruit are nutritious when they are fully ripened by sun and season, and not artificially stimulated. Nature knows what she is doing: she furnishes for every latitude the productions fittest for such latitude.

We need variety,—not at one time, but from time to time. The delicacies of the season will not hurt us; but the delicacies out of season certainly will, if long continued. The appetite so jaded as to crave oysters in July, or strawberries in December, needs careful correction by the adoption of the simplest habits. The palate naturally relishes what Nature has near at hand. A simple, pure life, gives appetite for pure and simple food.

---

Horse-Hair Snakes.—It is a common belief that the little snakes which are sometimes found in stagnant water, are vitalized horse-hairs. Of çourse this is incorrect. The creature is called the "fresh-water gordius," and its history is a very curious one. It is really a worm which lays about 8,000,000 eggs, and, did all these eggs come to maturity, our brooks and ponds would be full of them. As with the delicate eggs of many of the lower animals, comparatively few of those which are laid, survive, and these animals are very rare. They are hatched in the water from eggs, but after a time they leave the water; go on wet days into the grass; creep along the legs of torpid grasshoppers; enter their abdominal cavities, and undergo further transformation as parasitic worms. From this living prison they then escape, return to the water, and end their varied existence as the long thread-like worms which have been mistaken for living and moving hairs.

---

The best capital that a young man can start with in life is industry, good sense, courage, temperance, and an admiration for whatever is noble and good. They are better than cash, credit, or friends.

---

Marriage is the normal and legitimate order of society, and whatever tends to prevent marriage tends to destroy that order, to disrupt the true social bond, and to open the door for immorality.

